# Supplementary figures and images for: Describing the sounds of nature: Using onomatopoeia to classify bird calls for citizen science
Source: PLoS One. 2021 May 12;16(5):e0250363. doi: 10.1371/journal.pone.0250363 (PMC8115837; doi:10.1371/journal.pone.0250363)

**S1 Fig. Frequency chart for each bird call with confidence rating in brackets ( )**

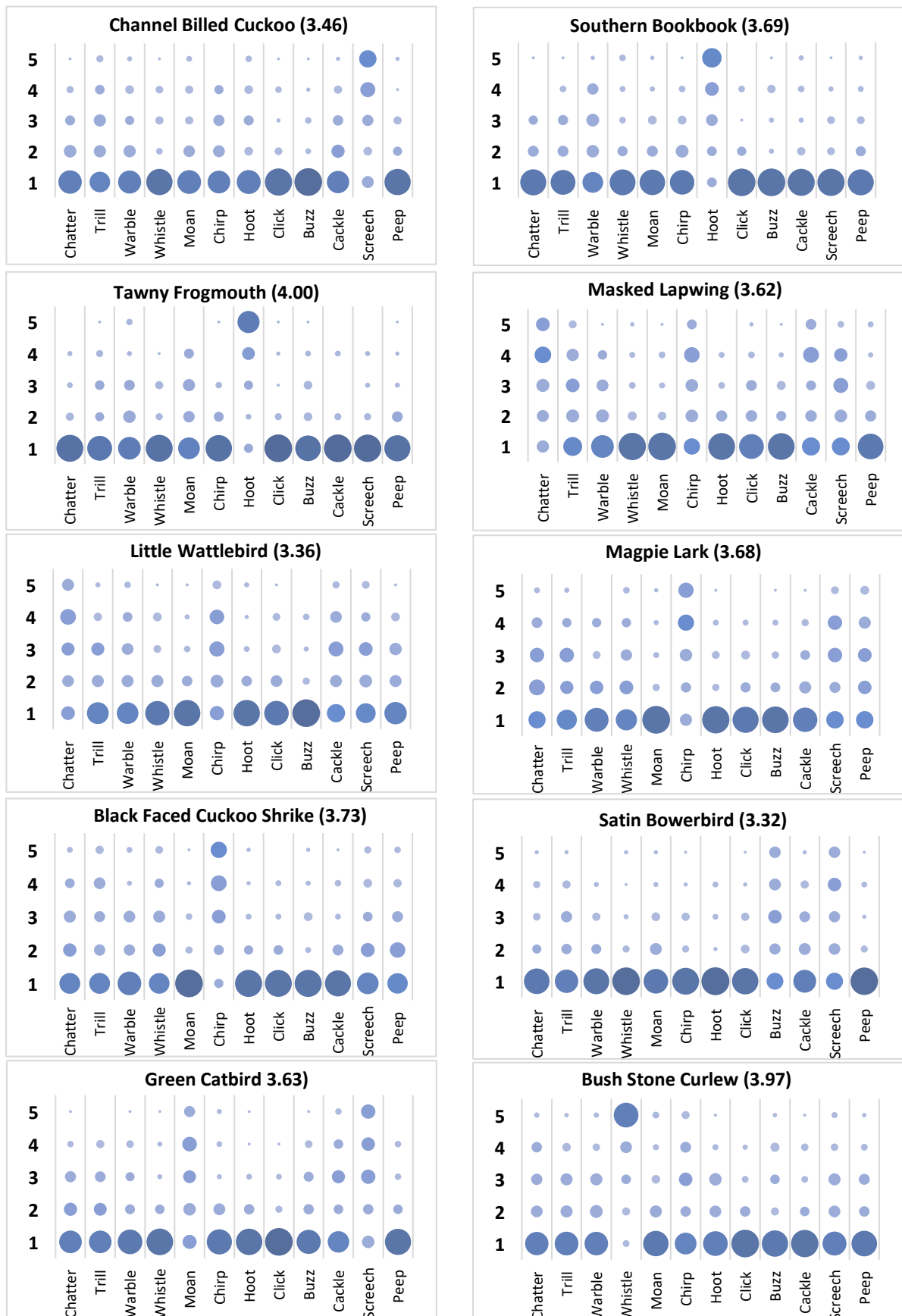

Supplement: S1 Fig — (PDF) [file pone.0250363.s003.pdf]
