# Supplementary material for: Describing the sounds of nature: Using onomatopoeia to classify bird calls for citizen science
Source: PLoS One. 2021 May 12;16(5):e0250363. doi: 10.1371/journal.pone.0250363 (PMC8115837; doi:10.1371/journal.pone.0250363)
Supplement: Attachment — Submitted filename: Response to reviewers.docx [file pone.0250363.s004.docx]

We thank the reviewers for their feedback and have made the following changes in response.

Reviewer #1:

Lines 234-240: We have made additions to the Procedure describing how the list of vocal descriptors was compiled including their sources, types of calls, reason for exclusion, and why we preferred to provide a list of descriptors rather than letting participants describe them. We highlight that we were seeking broadly applicable descriptors that were commonly used amongst people with an interest in bird calls.

Regarding the participants’ background information, we did not collect information regarding their first language or geographic location. Instead we now note that by including mTurk workers with a Masters rating, it was implied that they had a good grasp of English (added to line 261). However, lines 234-238 now acknowledge that we did not know if participants had English as a first language, and if they did not, how this may have impacted their ratings. As there is little research on this subject, we suggest the usefulness of future research contrasting participants who have the target language as a first or later language.

Regarding the distinction between semantics and onomatopoeia, we agree that we can’t know what someone’s conceptual construct of, for example, ‘whistle’ was. However, while this distinction is not the focus in the current study, this has been explored in other work (now described in lines 142-145). We also now discuss this distinction (lines 458-553) in relation to the provision of diverse types of information in audio categorisation tasks. Also, as noted in the Procedure, our choice of descriptors was due to seeking words commonly used to describe bird calls.

Reviewer #2:

We appreciate the need for consistency in terminology but have chosen to remove mention of ecoacoustics throughout, as this study is focused specifically on bioacoustics. In addition, we remove mention of ‘ecologists’ (line 95) as it was not our intention to contrast ecologists and the general public (which is how it read), but instead the different requirements for call identification in sound bite sharing and when using long-duration recordings of the environment. We make changes to lines 94-96 that we hope clarify this distinction.

Line 88: ‘Domain’ has been replaced with ‘environmental conservation’.

Obscure or unnecessary text has been changed or removed at lines 120-128 and 148-149.

We hope that these edits address the reviewers’ concerns.
